# Steal Phenomenon with Tonsillar Arteriovenous Malformation

**DOI:** 10.5811/cpcem.2019.5.42882

**Published:** 2019-07-01

**Authors:** Manish Amin, Krishan Chaddha, Phillip Aguìñiga-Navarrete, Sudha Challa, Madison B. Garrett

**Affiliations:** Kern Medical, Department of Emergency Medicine, Bakersfield, California

## Abstract

Cranial vascular malformations can cause symptoms of headache, stroke, transient ischemic attack, or other cerebrovascular disorders due to steal phenomenon. Subclavian steal phenomenon is a localized change in cerebral perfusion from a cranial arteriovenous malformation (AVM). We present the only recorded case of a tonsillar AVM causing a transient ischemic attack due to steal phenomenon.

## CASE PRESENTATION

A 35-year-old female presented to the ED with left arm and leg weakness. The patient had normal vital signs. Symptoms started 90 minutes before arrival. Past medical history included a questionable transient ischemic attack (TIA) two years prior. Physical examination noted enlargement of the right tonsillar region. The patient had four of five strength of the left upper and lower extremities with decreased light touch and pain sensation. Her National Institutes of Health Stroke Scale was one. Computed tomography (CT) of the brain was normal. CT angiogram of the brain and neck noted asymmetrical enlargement of the right pharyngeal tonsil associated with vessels and calcifications within the right tonsillar region (Image). Magnetic resonance imaging of the brain was normal. Neurology was consulted and the diagnosis of TIA from tonsillar arteriovenous malformation (AVM) was made.

## DISCUSSION

This is the only reported case of tonsillar AVM with an associated TIA. Venous malformations are common types of vascular malformations that present in infancy and expand throughout a patient’s lifespan. They typically present in a focal region, with 40% of them occurring in the head and neck.^1^ Vascular malformations noted within the cranium can contribute to symptoms of headache, stroke, TIA, or other cerebrovascular disorders.^2^ Intracranial AVMs are known to cause TIA symptoms due to steal phenomenon, which is a localized change in perfusion from an AVM. Use of antiplatelet agents in TIAs is evidence-based and reduces the possibility of recurrence of neurologic deficits in patients who have had TIAs. However, this poses a problem in patients known to have AVMs, as these agents can cause a higher rate of complications and rupture with AVMs.^3^

The steal phenomenon noted with intracranial AVMs has not been known to be associated with AVMs that are located outside the cranium. This may be an area of research for the future.

**CPC-EM Capsule**

What do we already know about this clinical entity?

It is understood that vascular malformations within the cranium can result in steal phenomenon, transient ischemic attack (TIA) and stroke-like symptoms.

What is the major impact of the image(s)?

This atypical case shows arteriovenous malformation in the palatine tonsillar region, where a correlation to steal phenomenon causing TIA symptoms is not well studied.

How might this improve emergency medicine practice?

This case adds to the differential diagnosis for stroke-type presentations.

## Figures and Tables

**Image:**
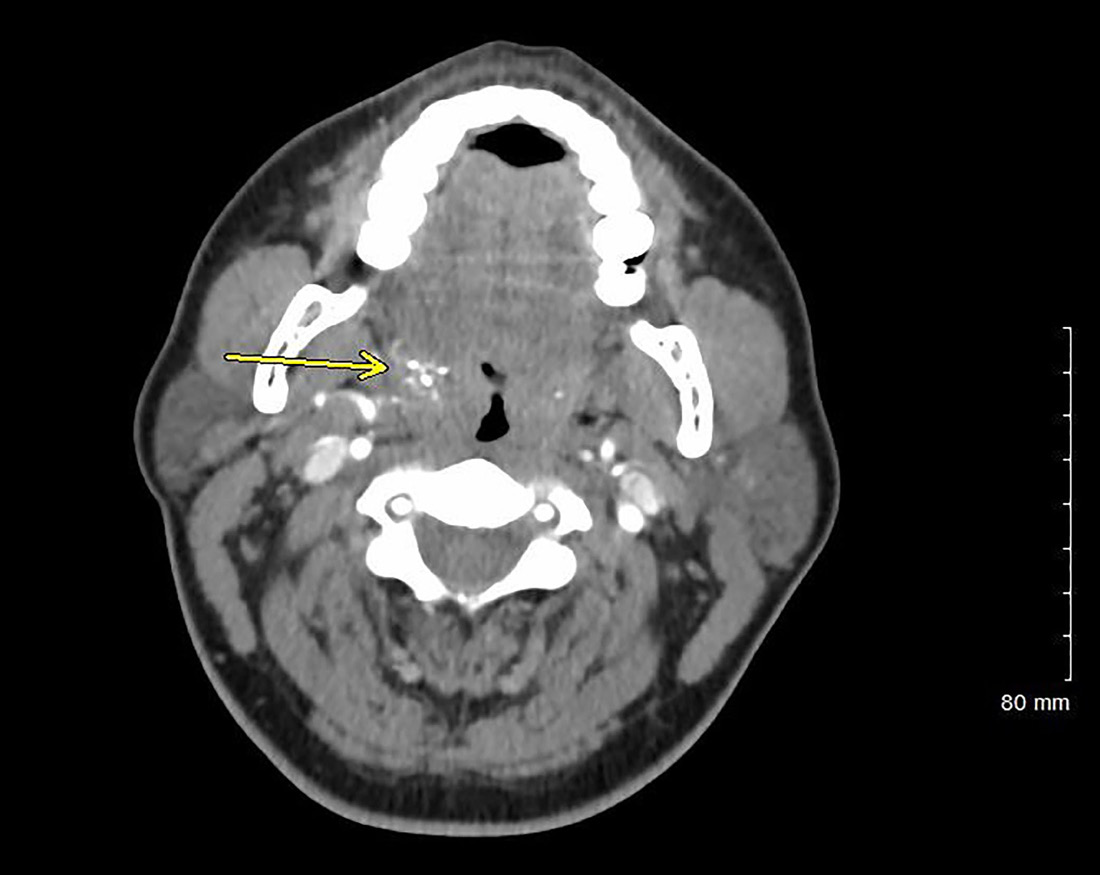
Axial computed tomographic angiography of the head showing palatine tonsillar arteriovenous malformation.
